# The attitude of online fans: perceived value, expectation, and identification on VAR satisfaction

**DOI:** 10.3389/fpsyg.2023.1288998

**Published:** 2024-01-05

**Authors:** Pei Deng, Weihua Yan, Ying Yu, Yeqin Zhang, Liqing Zhang

**Affiliations:** ^1^China Football College, Beijing Sport University, Beijing, China; ^2^China Institute for Advanced Olympic Studies, Beijing Sport University, Beijing, China; ^3^Sports Coaching College, Beijing Sport University, Beijing, China

**Keywords:** online fans, Video Assistant Referee, FIFA World Cup Qatar 2022, satisfaction, structural equation model, decision-aid technology

## Abstract

This study explores online fan satisfaction with the Video Assistant Referee (VAR) during the FIFA World Cup Qatar 2022. A structural equation model comprising perceived value, fan expectation, fan identification, and fan satisfaction was run. The online questionnaires were distributed among Chinese football fans. A total of 224 valid responses were received. Using indicators like Cronbach’s alpha coefficient, Kaiser-Meyer-Olkin (KMO) statistic, and Bartlett’s test of sphericity, the results were assessed for reliability, validity, and suitability. From the statistical results, the overall satisfaction of fans with VAR is the middle. Both fan expectation and perceived value positively affect satisfaction (*p* < 0.01); the path coefficients were 0.26 and 0.57. Contrastingly, fan identification exerts no significant effect on fan satisfaction (*p* > 0.05); and fan expectation indirectly affects fan satisfaction through perceived value (*p* < 0.01); the path coefficient was 0.29. The highest effect value for fan satisfaction is perceived value, followed by fan expectation. Consequently, to improve online fan satisfaction with VAR, researchers should focus on perceived value. This research contributes to a greater more comprehensive of Chinese online fans’ preference towards VAR at the FIFA World Cup Qatar 2022.

## 1 Introduction

The accelerated pace of the game and the variety of technical and tactical changes introduce novel challenges for referees to adjudicate. The referee may also fail to observe certain fouls due to the large size of the soccer field and the frequent and unpredictable nature of fouls. In addition, physical stress (Pizzera et al., 2022), noise (Unkelbach and Memmert, 2010) home advantage (Goumas, 2014), and the team’s reputation (Spitz et al., 2021; Pizzera et al., 2022) may also influence the decisions of referees. Mistakes, omissions, and misjudgments often occur in matches, often resulting to soccer referees are blamed and even abused by coaches, players, and fans. To optimize the game and promote fair play, the Fédération Internationale de Football Association (FIFA) has invested immense money and manpower into the referees’ team, and a steady stream of technology has been introduced in the game (FIFA, 2016). The application of Video Assistant Referee (VAR) was agreed upon by the International Football Association Board (IFAB) in 2016, and the technology was officially introduced during the 2018 World Cup held in Russia. As an auxiliary penalty technology, the introduction of VAR is considered a revolutionary process in soccer because it provides referees with more information and time, which exerts an impact on the accuracy of referees’ decisions, as well as on player’s foul behaviors, and fan’s watching experience (Errekagorri et al., 2020; Han et al., 2020; Lago-Penas et al., 2021; Kubayi et al., 2022; Zhang et al., 2022).

Fans are defined as individuals who believe that they are supporters of a team (Dietz-Uhler et al., 2000). Alternatively, fans can also be defined from the following perspectives: sports behavior, participation in sports events, and cognitive levels (Gantz and Wenner, 1991; Murrell and Dietz, 1992; Wann and Dolan, 1994). Fans, the main audience of soccer games, are quite vital for football tournaments (Karafil and Akgul, 2022).On one hand, the presence of fans at the game sets the atmosphere and provides motivation for football players who transform fans’ reactions for winning the game (Tamir, 2022); on the other hand, fans’ enthusiasm and satisfaction can contribute value for professional tournaments and professional soccer clubs (Uhrich and Benkenstein, 2010). For example, fans purchase tickets and team merchandize impact the profitability of tournaments and clubs. Major broadcasting platforms acquire broadcasting rights to attract a larger online audience, while brands are offered the right to display their logos and products or to operate stalls (i.e., a means of utilizing sponsorship). Thus, fans’ support and association of the sponsor’s brand contribute to the brand’s profitability (Bristow and Sebastian, 2001). Research indicates that an increasing emphasis on fan satisfaction within the sports industry (Grove et al., 2012; Sarstedt et al., 2014). Fan behaviors, such as sports attendance and loyalty, are significantly impacted by fan satisfaction (Trail et al., 2005; Shonk and Chelladurai, 2008), this highlights the importance of fan satisfaction in sports. According to FIFA financial reports, the largest share comes from the sale of television broadcasting rights (FIFA, 2023). Online fans, who watch matches either through television signals (cable operators or similar) or through live streaming (internet websites or platforms), play a crucial role. Therefore, ensuring the satisfaction of online fans is equally quite essential.

Although the significance of online fans, the number of studies on fan perceptions towards decision-aid technology in sports is limited (Hamsund and Scelles, 2021), and scientific research, which focuses merely on the satisfaction of online fans with VAR, remains limited. Notably, despite the FIFA World Cup Qatar 2022 was the best-equipped and largest event for VAR utilization, the satisfaction of online fans with VAR has not yet been investigated. Therefore, it is quite meaningful to analyze online fan satisfaction. The current study aims to analyze the satisfaction of online fans with VAR and the factors affect the satisfaction. Based on previous studies, we have selected perceived value, fan expectation, fan identification, and fan satisfaction as variables. Our hypotheses include perceived value, fan expectation, and fan identification have a significant impact on fan satisfaction, fan expectation positively influences perceived value, and fan expectation indirectly affects fan satisfaction through perceived value.

## 2 Literature review

### 2.1 Perceived value and fan satisfaction

Zeithaml (1988) defines perceived value as follows “Value is what you are paying for what you are getting “, and believes that the core of perceived value is the overall evaluation effected by individuals after comparing and weighing their perceived gain and perceived loss. Perceived value, as a variable, is often utilized in the analysis of service industries such as tourism and catering. In their analysis on the catering industry, Ge et al. (2021), Slack et al. (2021), and Tuncer et al. (2021) respectively noted that perceived value exerts an impact on satisfaction. Zeithaml (1988), via the psychology perspective, proves that consumers’ high perceived value of products is conducive to higher customer satisfaction. In sports, Cronin et al. (2000) studies on sports events indicate that perceived value exerts a positive impact on satisfaction. In the research on the satisfaction of tennis event spectators, it is observed that the enjoyment and economic perceived value of the spectators exerts a positive impact on satisfaction (Wang et al., 2021). Calabuig-Moreno et al. (2016) conducted a study utilized a basketball club in the Spanish League to investigate the satisfaction of 563 spectators to the game, and they noted that the perceived value positively affects the satisfaction of the audience. Similarly, the research on basketball audience satisfaction indicates that perceived value is the most influential factor in sports audience satisfaction (Moreno et al., 2015) and Mostaghimi et al. (2016) obtained similar conclusions.

Based on the aforementioned research, the following hypothesis is proposed:

*H1*: Perceived value positively affects fan satisfaction.

### 2.2 Fan expectation and fan satisfaction

The variables in the Swedish Customer Satisfaction Barometer (SCSB), the American Customer Satisfaction Index (ACSI), and the European Customer Satisfaction Index model (ECSI) all contain customer expectations; Tong et al. (2022) noted that those public expectations directly influence public satisfaction in their investigation of Chinese residents’ contentment with community health education. In the consumer behavior field, based on the expectation theory proposed by Oliver (1980), numerous studies explore the relationship between consumer expectation and satisfaction. Cardozo (1965) noted that consumer satisfaction is lower when products do not meet consumer expectations. Research on customer satisfaction in the Iranian automobile market indicated customer expectations affect perceived value and satisfaction (Mostaghimi et al., 2016). Another researcher based on the ACSI analyze community health education indicated the expectation indirectly affects fan satisfaction through perceived value (Tong et al., 2022).

Based on the above research, the following assumptions are made:

*H2*: Fan expectation positively influences perceived value.

*H3*: Fan expectation positively affects fan satisfaction.

*H4*: Fan expectation indirectly affects fan satisfaction through perceived value.

### 2.3 Fan identification and fan satisfaction

In the social identity theory, individuals who become part of a group gain a sense of belonging and create more emotional connections. When constructing the Sports Spectator Satisfaction Model (SSSM), Van Leeuwen et al. (2002) indicated that fans with higher identification exhibit higher satisfaction than those with lower identification. Fans with a higher degree of identification concentrate more on the match and its result. In football, if a fan exhibits a higher identification with a certain team, they usually display behaviors and attitudes that are in the best interests of the team (Masayuki et al., 2015). However, Winand et al. (2021) observed that for a team, the higher the degree of identification, the lower the satisfaction with VAR. The utilization of VAR exerts a negative impact on the competition and athletes. Football fan has emotional connection with a club, the stronger the identification with the team by the fans, the more likely they are to be committed to safeguarding its best interests (Winand and Fergusson, 2018).

Therefore, this study raises the following assumption:

*H5*: Fan identification negatively affects fan satisfaction.

**Figure 1:**
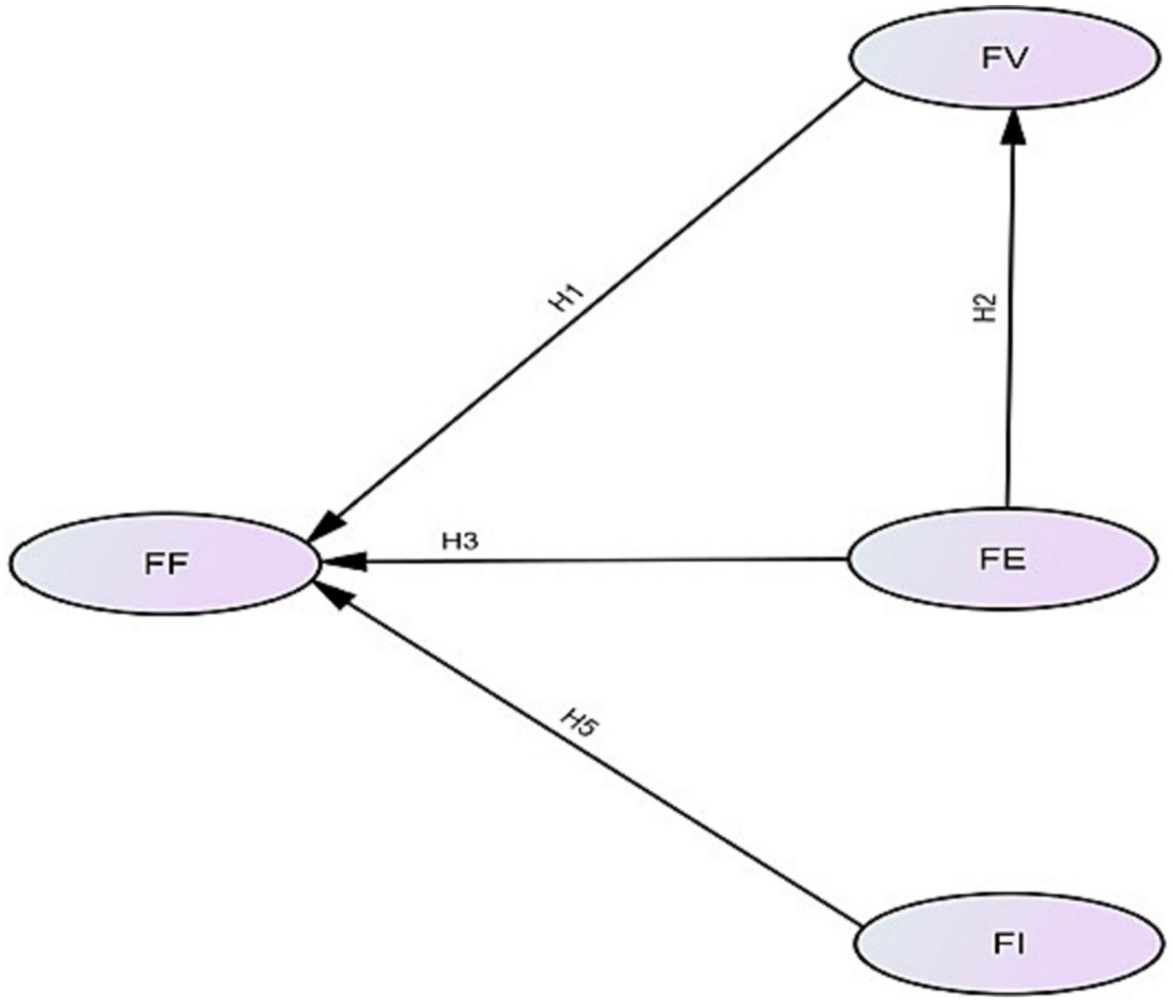
Hypothetical relationship model of online fan satisfaction with VAR.

In summary, the current study develops a structural equation model of fan satisfaction with VAR, which is depicted in Figure 1.

## 3 Methods

### 3.1 Questionnaire design

Based on previous studies (Bauer et al., 2008; Peng et al., 2019; Luan and Zhang, 2020; Winand et al., 2021), a designed scale was utilized to evaluate the perceived value, expectation, identification, and satisfaction of fans. The fan satisfaction measurement was adapted from Winand et al. (2021), and included comments such as “I agree with the utilization of VAR in FIFA World Cup matches” and “I am satisfied with the utilization of VAR at the FIFA World Cup Qatar 2022.” The assessment of perceived value was adapted from Peng et al. (2019), and it included questions such as “I think VAR has enhanced the quality of football matches at the FIFA World Cup Qatar 2022,” “I think VAR has enhanced the atmosphere of the games,” and “I think the utilization of VAR in FIFA World Cup Qatar 2022 has instilled a sense of pleasure in me.” In the main section of the questionnaire, fans’ opinions were collected in the form of a 5-point Likert scale, with “1″ representing not satisfied at all, “5″ representing quite satisfied, and “3″ representing average. The questionnaire was distributed to fans via an online format.

### 3.2 Selection of variables

Based on the evaluation of fan satisfaction, the data pertaining to each variable were tested for Kaiser–Meyer–Olkin (KMO) and Bartlett’s sphericity (KMO = 0.864), and the approximate chi-square value was large with *p* < 0.001 (Table 1). This result indicated that the data were suitable for factor analysis. When extracting the common factors, it was observed that all common factors exhibit >0.7 Cronbach’s Alpha. Thus, 4 latent variables were suitable, along with 13 measurement indicators.

**Table 1:** Test results of Kaiser–Meyer–Olkin and Bartlett on theoretical indicators.

KMO	Bartlett’s sphericity test		
	Approximate chi-square value	Df	*p* value
0.846	1402.209	78	<0.001

### 3.3 Data collection and analyses

The questionnaire was distributed between November 20 and December 18, 2022. Wjx, an online survey system, was utilized to construct and perform the survey. The questionnaires were distributed using a convenience sample among Chinese football fan, receiving a total of 245 responses. After excluding the ones with an exceedingly short answering time and high consistency of answers, 224 valid questionnaires were collected, and a 91.42% recovery rate was observed. Herein, reliability and validity were tested using the statistical software IBM SPSS version 26.0 (IBM Corp., Armonk, NY), and using Cronbach’s alpha coefficient, the questionnaire reliability was assessed. The principal component analysis (PCA) and varimax-rotation methods were employed for conducting factor analysis; using the Kaiser-Meyer-Olkin (KMO) statistic, Bartlett’s test of sphericity, and the cumulative variance, the questionnaire’s validity was assessed. The assessment validity for each dimension involved composite reliability and the average variance extracted. To judge the model adaptation, the normed chi-square (CMIN/DF), goodness-of-fit index (GFI), adjusted goodness-of-fit index (AGFI), root mean square error of approximation (RMSEA), normed fit index (NFI), and Tucker–Lewis Index (TLI), comparative fit index (CFI), normed-fit index (NFI), parsimonious normed-fit index (PNFI), parsimonious goodness-of-fit index (PGFI), and parsimonious comparative fit index (PCFI) were considered. Using Bootstrap sampling, the coefficient product method was applied to test for mediating effects; 2,000 replicate samples were selected with 95% confidence intervals,

## 4 Results

The results of the reliability and validity tests exhibited a Cronbach’s Alpha coefficient greater than 0.7 indicating that the questionnaire possessed more optimal internal consistency. Table 2 indicates the Alpha coefficients of the four latent variables in the 0.752–0.856 scale range. Additionally, the Alpha coefficient for the overall questionnaire is 0.842, indicating good reliability of the scale (Table 2).

**Table 2:** Results of confirmatory factor analysis.

Latent variable		FL	Cronbach’ α	AVE	CR
Summary table			0.842		
Perceived value	FV1	0.786	0.845	0.647	0.846
	FV2	0.803			
	FV3	0.824			
Fan expectation	FE1	0.796	0.836	0.639	0.841
	FE2	0.873			
	FE3	0.722			
Fan identification	FI1	0.734	0.752	0.503	0.752
	FI2	0.699			
	FI3	0.694			
Fan satisfaction	FF1	0.744	0.845	0.619	0.865
	FF2	0.822			
	FF3	0.889			
	FF4	0.676			

The principal component analysis (PCA) and varimax-rotation methods were utilized for factor analysis, a total of four common factors were extracted, and the explained variance rate attained 73.36%. The factor loading matrix after conducting the varimax-rotation method is illustrated in Table 3. Factor scores for the selected indicators were all higher than 0.6, which indicates that the observed variables can effectively reflect the four latent variables of perceived value, fan expectation, fan identification, and fan satisfaction. CR is another method of determining the questionnaire’s reliability (Wang et al., 2022). The reported CR was higher than 0.70, which indicated satisfactory reliability. Factor loading and the AVE of each variable are usually utilized to evaluate the validity of the scales. The test result indicated that factor loading was higher than 0.5 and that the AVE exceeded 0.5 (Mia et al., 2022; Zhao et al., 2022), which indicates that the questionnaire exhibits satisfactory validity.

**Table 3:** Factor loading matrix.

Variable	component			
	1	2	3	4
FV1	0.685			
FV2	0.885			
FV3	0.818			
FE1		0.820		
FE2		0.834		
FE3		0.824		
FI1			0.824	
FI2			0.811	
FI3			0.814	
FF1				0.726
FF2				0.876
FF3				0.849
FF4				0.614

Discriminant validity among the latent variables can be established when the AVE of each variable is greater than the correlation coefficients between variables after considering the square root. Table 4 indicates that the AVE for perceived value, fan expectation, fan identification, and fan satisfaction are all greater than the inter-dimensional correlation coefficients, which indicates satisfactory discriminant validity between the four dimensions of the scale.

**Table 4:** Discriminant validity analysis.

	Perceived value	Fan expectation	Fan identification	Fan satisfaction
Perceived value	0.804			
Fan expectation	0.434	0.799		
Fan identification	0.074	0.081	0.709	
Fan satisfaction	0.627	0.502	0.081	0.787

**Figure 2:**
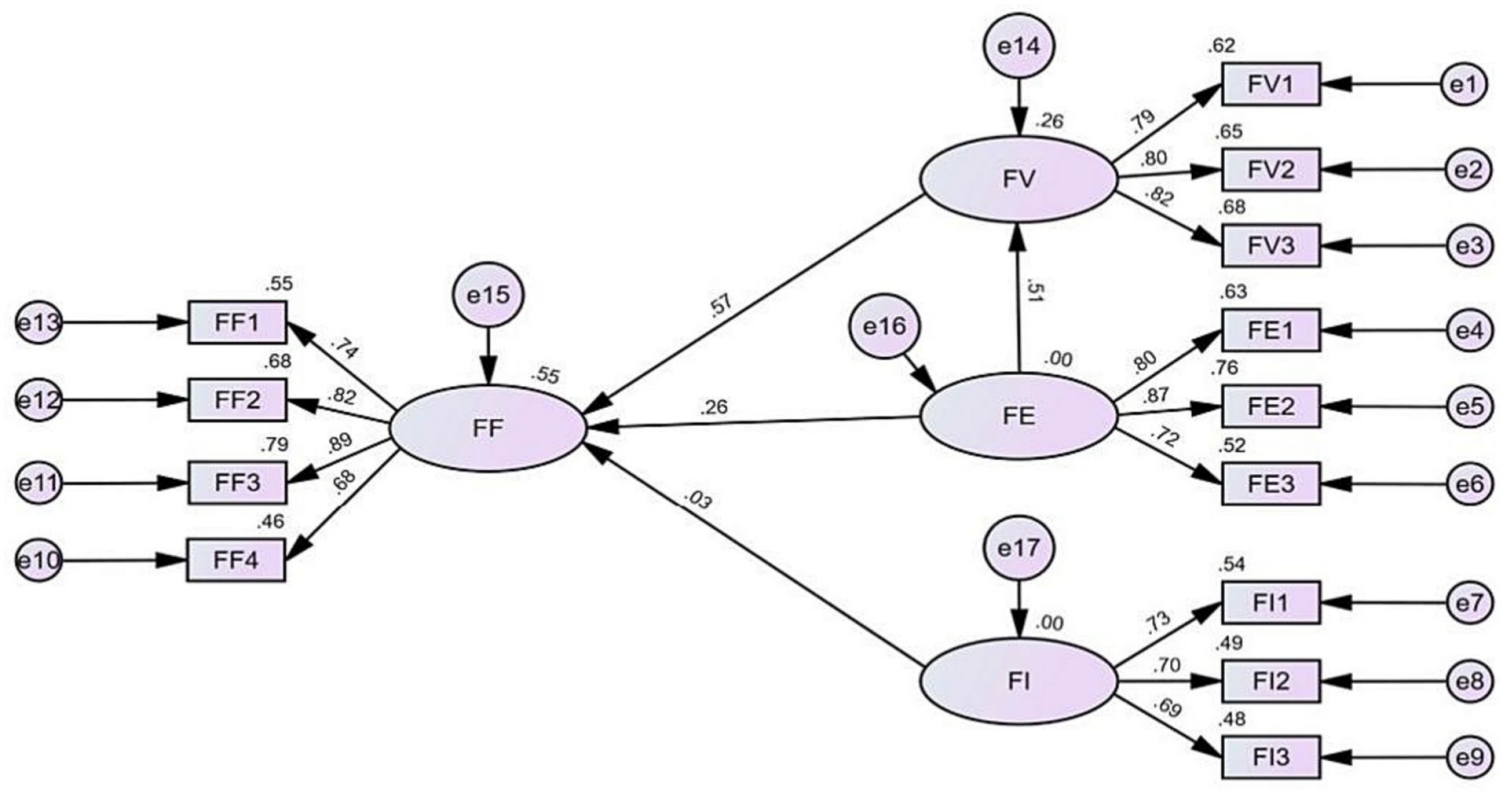
Structural equation modeling of online fan satisfaction.

The test results of model adaptation exhibited good fit (Table 5), which indicates that the fit indexes meet the criteria of SEM studies. This study utilizes four questions to measure fan satisfaction, with the following means: “FF1”(*M* = 4.41), “FF2”(*M* = 3.68), “FF3”(*M* = 3.70), and “FF4”(*M* = 3.61); as illustrated in Table 6; Figure 2 indicate that perceived value and fan expectation impact fan satisfaction (*p* < 0.01), and that fan expectation impacts perceived value (*p* < 0.01). Thus, research hypotheses H1, H2, and H3 are supported. However, because the impact of fan identification on fan satisfaction has not attained a statistically significant level (*p* > 0.05). Therefore, Hypothesis H5 is not supported. The confidence interval for the results of the mediated path analysis was [0.173 ~ 0.465], with a confidence interval not containing 0, an effect value of 0.293, and the value of *p* less than 0.01, which indicates a significant effect of the mediated path. The path coefficient results are depicted in Table 7. Thus, research hypothesis H4 is supported.

**Table 5:** Fitting coefficients of the model indicators.

	Judgment standard	Measure value	Acceptance level
CMIN/df	1–3	2.093	Good
GFI	>0.900	0.916	Very good
AGFI	>0.800	0.875	Good
RMSEA	<0.080	0.070	Good
NFI	>0.900	0.911	Very good
TLI	>0.900	0.937	Very good
CFI	>0.900	0.951	Very good
PGFI	>0.500	0.614	Good
PNFI	>0.500	0.713	Good
PCFI	>0.500	0.744	Good

**Table 6:** Model path coefficient of structural equation model and hypothesis test results.

The path	Unstandardized coefficient	standardized coefficient	SE	CR	*p*	results
H1 FE → FV	0.514	0.599	0.081	6.429	***	support
H2 FE → FF	0.259	0.278	0.098	6.086	***	support
H3 FV → FF	0.569	0.523	0.083	3.337	***	support
H5 FL → FF	0.022	0.020	0.057	0.441	0.725	nonsupport

***Significance level < 0.01.

**Table 7:** Effects test.

The path	effects	*p*	Standard error	95%CI
FE → FF	0.570	***	0.093	0.372 ~ 0.739
FE → FV	0.514	***	0.092	0.328 ~ 0.686
FV → FF	0.260	***	0.098	0.074 ~ 0.449
FE → PV → FF	0.293	***	0.074	0.173 ~ 0.465

***Significance level < 0.01.

## 5 Discussion

This study employed a structural equation model to analyze online fan satisfaction with the utilization of VAR at the FIFA World Cup Qatar 2022. From the statistical results, the overall satisfaction of fans with VAR assumes the middle value. The fitting results of the structural equation model indicated that perceived value and fan expectation exert a positive effect on satisfaction, and that fan expectation indirectly affects fan satisfaction through perceived value. In addition, the highest effect value for fan satisfaction is perceived value, followed by fan expectation. However, fan identification does not exert a significant effect on satisfaction.

The majority of fans agree with the utilization of VAR because it generally exerts a positive impact on the game: it enhances the accuracy of penalties and reduces malicious fouls (Goumas, 2014). The statistics indicated that fan satisfaction was average, and Winand et al. (2021), who explored the satisfaction of German and English fans with VAR, also noted that the overall satisfaction of fans was average, with the satisfaction scale data exhibiting neutral values. This observation may be rationalized as follows: the utilization of VAR has exerted numerous negative effects on the game. The number of game interruptions increased, which exerted a negative impact on athlete status (Carlos et al., 2019; Han et al., 2020). In addition, fans exhibit an emotional connection with their favorite team and may project, deny, or transfer the emotion of the team’s goal and result of the game to other factors. With regard to fans, VAR is an emotion transfer object, which also affects VAR satisfaction. In Bertin et al. (2023)’s study, it was also noted that negative tweets on VAR increased at the end of the group stage, which may be related to the elimination of the team, and the fans expressed negative emotions on VAR to rationalize the situation of the team and maintain the image of the team.

The higher the VAR fan perception value, the higher the actual satisfaction of the fan. Perceived value was identified as the most influential variable in all studies, herein, the results of this study are similar to those of previous ones (Greenwell et al., 2002; Moreno et al., 2015; Calabuig-Moreno et al., 2016). Using the analysis path, it was noted that the fan’s perceived value of VAR in enhancing the quality and atmosphere of the game exerted a marginal impact on VAR satisfaction. This observation may be rationalized as follows: fans usually discuss controversial events during the match, and the heated discussion of fans on match events impacts the match atmosphere. The application of VAR can provide fans with correct penalty results, which reduces the opportunity for fans to discuss (Winand et al., 2021); thus, fans exhibit less perception of the enhanced match atmosphere after VAR intervention. Therefore, the impact on satisfaction is less considerable. Second, the most direct perception of online fans after the intervention of VAR is as follows: it interrupts the game and affects its fluency. Therefore, the perceived value of enhancing the quality of the competition is limited, and so is the impact on satisfaction is smaller.

The higher the fans’ sense of acquisition after the application of VAR, the higher the evaluation of VAR. Therefore, it is proposed that the VAR equipment be optimized, which can not only provide referees with clearer videos but also enhance the comfort of online fans watching matches when VAR intervenes. The FIFA World Cup Qatar 2022 stadiums are considered to be an effective method of increasing fan satisfaction with VAR by providing timely feedback (Hamsund and Scelles, 2021). Thus, it is recommended that major leagues provide fans with the referee review screen and report the results on the big screen and the live broadcast platform to enhance the fans’ watching experience. In addition, leagues should optimize the selection of referees, unify the penalty scale, and enhance the workability of referees; thus, the number of unnecessary interventions can be reduced.

According to the research results, fan expectation can directly and positively affect fan satisfaction, and Tong et al. (2022) also arrived at this conclusion. When testing the intermediary path, it is observed that fan expectation can also indirectly affect fan satisfaction by influencing fan perceived value (Mostaghimi et al., 2016; Tong et al., 2022). The aforementioned observation may be rationalized as follows: expectation is considered a cognitive basis in the evaluation of satisfaction of fans (Cardozo, 1965). Fan expectation of VAR is based on fans’ understanding of the application rules, application value, and VAR concept. “Minimum intervention, maximum benefit” idealizes the VAR concept. The utilization of VAR is not aimed at correcting all wrong decisions, nor pursuing a 100% accuracy level for all decisions. VAR should ideally enhance the fairness of the game; to avoid a scenario in which the game is excessively interrupted, VAR intervenes against “clear and apparent errors” or “seriously missed incidents” within the rules that change the course of the game (IFAB, 2019). However, fans will be unsatisfied if VAR is not conducive to the team they support or does not intervene in the events of the match that fans feel should be reviewed. Thus, to enhance fan satisfaction, publicity efforts should be increased, and fan expectations on the utilization of VAR should be appropriately guided. The promotion of the use of VAR should not only show fans the rationality of the existence of VAR and the process of using VAR, but should also promote the concept of VAR, show the improvement of VAR, the beneficial effects of the use of VAR on the game. Then promote the fans to further understand VAR in order to create reasonable expectations.

Through the path coefficient analysis, it is noted that fan identification exerts no significant impact on fan satisfaction. This research result is consistent with that of Winand and Fergusson (2018) who considered goal-line technology (GLT); however, it is inconsistent with that of Winand et al. (2021) who analyzed VAR. This observation is mainly attributable to the following: the objects investigated herein are mainly Chinese online fans, the China National Football Team did not participate in the FIFA World Cup Qatar 2022. As a major factor affecting the identity of football fans, compared with the local fans of each participating team, Chinese fans lack a sense of belonging and local identity (Wann and Tucker, 1996). When the team changes the score or wins the game with VAR, football fans lack extreme excitement, happy emotions, and identity pride. Similarly, when VAR is involved, losing a match to an opponent or lagging behind can only lead to temporary disappointment and anger from fans (Dong-Kyu, 2020). Thus, the following relationship is observed: fan identification exerts no significant impact on fan satisfaction, which is inconsistent with the findings of Winand et al. (2021).

## 6 Conclusion

The satisfaction and feelings of fans must be highlighted because they crucially influence the development of the football economy and sports industry. Based on the common indicators associated with satisfaction models in the marketing field, and on the theoretical reference pertaining to previous studies, this study constructed a VAR-based fan satisfaction model and is the first to investigate Chinese football fans’ VAR perception. The study selected perceived value, fan expectations, fan identification, and satisfaction as the variables for the structural equation model. It proposes that the overall satisfaction of fans with VAR assumes the middle value. The study indicated that fan expectation and perceived value positively affect fan satisfaction and that perceived value exerts an intermediary role between fan expectation and fan satisfaction, whereas fan expectation indirectly affects fan satisfaction through perceived value. To enhance fan satisfaction and, enhancing the public image of VAR, publicity efforts should be increased, and fan expectations on the utilization of VAR should be appropriately guided.

## 7 Limitations and suggestions for future research

Even if the proposed model is tested, some limitations persist. The questionnaire is issued to Chinese fans, and the teams participating in the World Cup are mostly distributed in various countries; therefore, the satisfaction of fans in different countries and regions with the World Cup VAR can be compared. In addition, some studies have indicated that the utilization of VAR in crucial moments of the game has an impact on match results (Kolbinger and Knopp, 2020; Bertin et al., 2023), and that the number of tweets on negative emotions associated with VAR increases. Therefore, more variables can be added to future research on fan satisfaction with VAR.

## Data availability statement

The raw data supporting the conclusions of this article will be made available by the authors, without undue reservation.

## Ethics statement

Ethical review and approval was not required for the study on human participants in accordance with the local legislation and institutional requirements. Written informed consent from the patients/ participants was not required to participate in this study in accordance with the national legislation and the institutional requirements.

## Author contributions

PD: Writing – original draft. WY: Writing – review & editing. YY: Data curation, Writing – review & editing. YZ: Resources, Writing – review & editing. LZ: Supervision, Writing – review & editing.
